# Ornithine-δ-aminotransferase is essential for Arginine Catabolism but not for Proline Biosynthesis

**DOI:** 10.1186/1471-2229-8-40

**Published:** 2008-04-17

**Authors:** Dietmar Funck, Bettina Stadelhofer, Wolfgang Koch

**Affiliations:** 1 Department of Plant Physiology and Biochemistry, Biology Section, University of Konstanz, Universitätsstraße 10, 78464 Konstanz, Germany; 2 ZMBP Plant Physiology, University of Tübingen, Auf der Morgenstelle 1, 72076 Tübingen, Germany

## Abstract

**Background:**

Like many other plant species, Arabidopsis uses arginine (Arg) as a storage and transport form of nitrogen, and proline (Pro) as a compatible solute in the defence against abiotic stresses causing water deprivation. Arg catabolism produces ornithine (Orn) inside mitochondria, which was discussed controversially as a precursor for Pro biosynthesis, alternative to glutamate (Glu).

**Results:**

We show here that ornithine-δ-aminotransferase (δOAT, At5g46180), the enzyme converting Orn to pyrroline-5-carboxylate (P5C), is localised in mitochondria and is essential for Arg catabolism. Wildtype plants could readily catabolise supplied Arg and Orn and were able to use these amino acids as the only nitrogen source. Deletion mutants of δ*OAT*, however, accumulated urea cycle intermediates when fed with Arg or Orn and were not able to utilize nitrogen provided as Arg or Orn. Utilisation of urea and stress induced Pro accumulation were not affected in T-DNA insertion mutants with a complete loss of δ*OAT *expression.

**Conclusion:**

Our findings indicate that δOAT feeds P5C exclusively into the catabolic branch of Pro metabolism, which yields Glu as an end product. Conversion of Orn to Glu is an essential route for recovery of nitrogen stored or transported as Arg. Pro biosynthesis occurs predominantly or exclusively via the Glu pathway in Arabidopsis and does not depend on Glu produced by Arg and Orn catabolism.

## Background

Amino acids are required for protein biosynthesis, but have also additional functions like nitrogen storage and transport. Proline (Pro) and the non-proteinogenic γ-aminobutyrate are also used as compatible osmolytes that are accumulated by many plant species in response to water deprivation [1]. Arginine (Arg) and Arg-rich proteins serve as an important storage form of organic nitrogen in many plants, especially in seeds [2-4]. Additionally, Arg or ornithine (Orn) are the precursors for the synthesis of spermine, spermidine and related polyamines, which are essential for sexual reproduction and additionally play important roles in stress tolerance [5,6]. Therefore, biosynthesis and degradation of amino acids is embedded in a complex metabolic and regulatory network that allows the plant to serve all the requirements of growth and environmental adaptation.

The primary pathways for amino acid biosynthesis and degradation in plants were mainly deduced by identifying genes or enzyme activities homologous to prokaryotic or fungal model systems. However, the localisation of metabolic pathways in different compartments within the plant cell is still not satisfyingly clarified [7]. Additional complications arise from the possibility of substrate channelling in multi-enzyme complexes that could separate individual pathways despite the use of common metabolites.

Arg biosynthesis seems to be localised predominantly in plastids, with some ambiguous localisation prediction of enzymes in the cytosol [3]. Arg decarboxylases (ADC1 & 2), the committing enzymes for polyamine synthesis in Arabidopsis have a predicted localisation in the cytosol or chloroplast (SubCellular Proteomic Database [8]), whereas Arg catabolism takes place in mitochondria via arginase [9]. Arginase produces urea, which is further degraded by urease in the cytoplasm, and Orn, which could be exported from mitochondria to re-enter Arg biosynthesis [10]. Two transporters for basic amino acids that could mediate exchange of Arg and Orn across the mitochondrial inner membrane have been identified by complementation of a yeast Arg11 mutant [11,12].

Pro is mainly synthesised in the cytosol from glutamate (Glu) via pyrroline-5-carboxylate (P5C) by the sequential action of P5C synthetase (P5CS) and P5C reductase (P5CR). In Arabidopsis, two isoforms of P5CS are present, with P5CS2 as a housekeeping isoform and P5CS1 being responsible for the accumulation of Pro in response to stress [13,14]. In response to osmotic stress, P5CS1 becomes re-localised to plastids [14]. For degradation, Pro is imported into mitochondria where it is converted back to Glu by Pro-dehydrogenase (ProDH) and P5C-dehydrogenase (P5CDH) [15,16]. There is also evidence for a pathway of Pro synthesis from Orn, and Orn-δ-aminotransferase (δOAT) has been implicated in this pathway [17]. δOAT transfers the δ-amino group of Orn to α-ketoglutarate or related α-keto acids, thereby forming glutamate-5-semialdehyde (GSA) and Glu. The equilibrium of this reaction was found far on the GSA/Glu side [17]. GSA is in spontaneous equilibrium with the cyclic P5C, which is the common intermediate in Pro biosynthesis and degradation. Formation of GSA/P5C from Orn was postulated to constitute an alternative pathway of Pro synthesis and accumulation, with Arg or Orn instead of Glu as precursors [18].

The first gene encoding a plant δOAT was cloned from a moth bean cDNA library by functional complementation of an *E. coli *Pro-auxotroph strain deficient in the conversion of Glu to P5C [18]. Sequence similarity to mammalian and bacterial enzymes strongly suggested that the gene encoded a δOAT rather than an αOAT. Recently, heterologous expression of the moth bean δOAT in *E. coli *revealed that its activity was inhibited by serine, isoleucine and valine, but not Pro [19]. The Arabidopsis *δOAT *gene (At5g46180) was identified by sequence comparison and was found to be upregulated in young seedlings and in response to salt stress [20]. However, out of eleven prediction programs for subcellular localisation including mitochondria, all strongly predict a targeting of the δOAT protein to mitochondria, with a putative transit peptide cleavage site after Phe16 [21,22]. Targeting δOAT to mitochondria strongly suggests that P5C is fed into the Pro degradation pathway rather than into Pro biosynthesis. Additionally, radiotracer experiments with externally supplied Orn indicated that Pro formed from Orn preserves the δ-amino group, whereas the α-amino group is lost [23]. The latter results suggested that Orn to Pro conversion proceeds via an αOAT.

On the other hand, transgenic tobacco and rice plants overexpressing the Arabidopsis *δOAT *gene had increased Pro content and increased stress tolerance, supporting the concept that Orn conversion can contribute to Pro accumulation [24,25]. Use of gabaculine as a potent inhibitor of δOAT suggested that in radish cotyledons Orn conversion could contribute to salt-induced Pro accumulation, whereas in rice leaves this pathway was probably of minor importance or not at all active [26,27]. None of the studies published at present directly investigated the subcellular localisation of δOAT or provided strong evidence for a physiological function of δOAT in Pro synthesis in non-transgenic plants.

In the present study we have analysed the physiological function of δOAT in Arabidopsis. We provide experimental confirmation of the predicted localisation of δOAT in mitochondria using a δOAT-GFP fusion protein. With the use of loss-of-function T-DNA insertion mutants we demonstrate that δOAT is essential for nitrogen recycling from Arg, whereas it does not seem to contribute to Pro biosynthesis.

## Results

### Ornithine-δ-aminotransferase is localised in mitochondria

**Figure 1:**
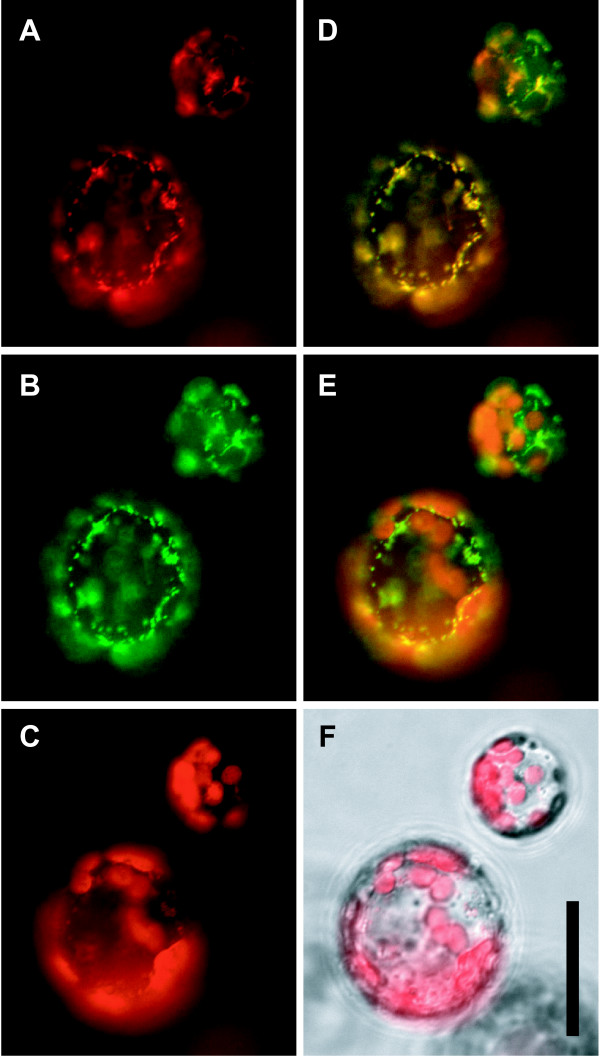
**δOAT is localised in mitochondria**. Leaf protoplasts from Arabidopsis plants stably transformed with a δ*OAT-GFP *fusion construct under control of the CaMV 35S promoter. **A**: Fluorescence signal of MitoTracker orange; **B**: GFP signal; **C**: Autofluorescence of chlorophyll; **D**: Merge of A and B; **E**: Merge of B and C; **F**: Merge of C and a brightfield image. Scale bar = 20 μm.

As a first step to determine the physiological function of δOAT we determined the subcellular localisation of the enzyme. We fused the cDNA of *δOAT *in frame to the N-terminus of GFP and expressed the fusion protein in Arabidopsis and in *Nicotiana benthamiana*. Intact cells and protoplasts from stably transformed Arabidopsis plants or from transiently transformed *N. benthamiana *leaves showed a clear punctate distribution of δOAT within the cells (Fig. 1, Additional file 1, and data not shown). Staining of leaf sections with MitoTracker was not successful, therefore double labelling was performed on protoplasts. In protoplasts, colocalisation of the GFP-signal with the orange fluorescence of MitoTracker clearly identified the δOAT-GFP containing compartments as mitochondria, confirming the sequence-based prediction of subcellular localisation.

### Ornithine-δ-aminotransferase does not contribute to stress-induced proline accumulation

**Figure 2:**
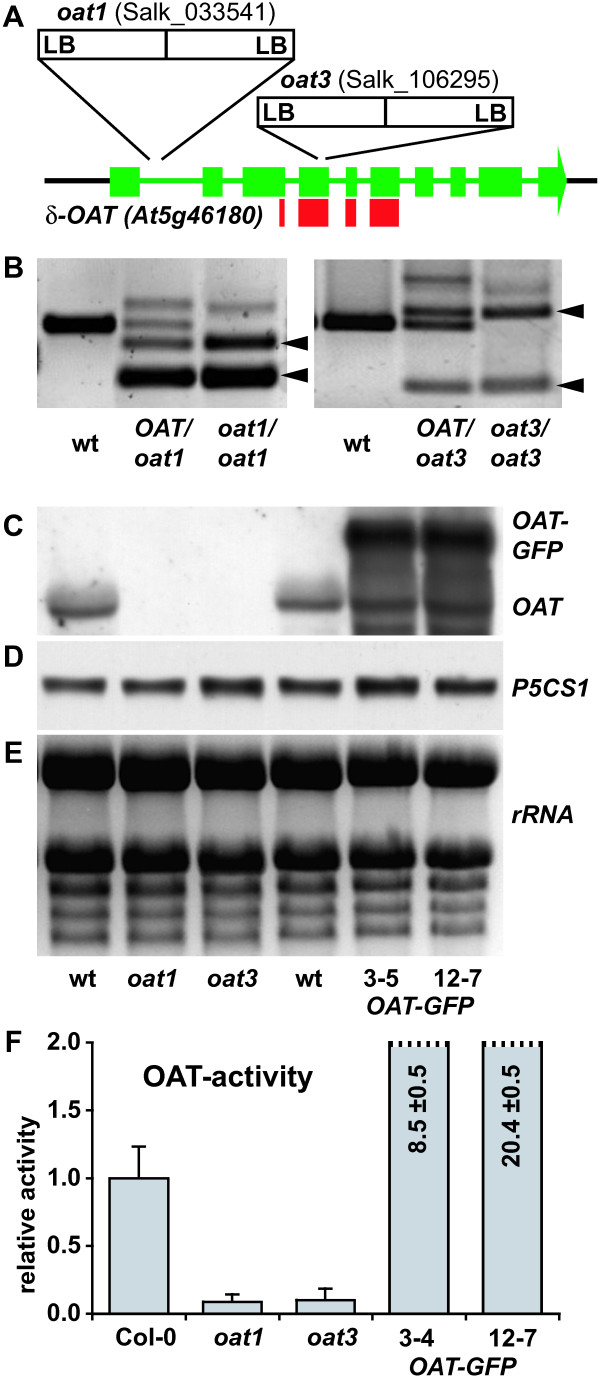
**Molecular and biochemical characterisation of *oat*-knockout mutants**. **A**: Schematic representation of the exon-intron structure of δ*OAT *(At5g46180) with the T-DNA insertion points in *oat1 *and *oat3*. Thick green bars indicate exons, thin green bars indicate introns. The thick red bars indicate the part of the mRNA used as probe for northern blotting. **B**: PCR with two gene-specific primers and one primer complementary to the T-DNA left border identified homozygous plants. Appearance of two T-DNA specific bands (indicated by arrowheads) indicated an inverted tandem repeat of the T-DNA. **C**: Northern blot with the δ*OAT*-specific probe on wildtype, *oat *mutants and δ*OAT-GFP *transgenic plants. **D**: The same membrane re-probed with a *P5CS1*-specific probe. **E**: EtBr staining of the corresponding RNA-gel to demonstrate equal loading. **F**: OAT activity in whole plant extracts. OAT activity is expressed in arbitrary units of P5C produced per mg total protein during 20 min. Error bars indicate SD of triplicate assays, the whole experiment was repeated with similar results from independent samples.

The mitochondrial localisation of δOAT indicated that it is not involved in the formation of Pro, since a reversed reaction of ProDH is energetically unfavourable. Due to the chemical instability of GSA/P5C, an export of this intermediate to the cytosol and thus a contribution to Pro synthesis appears rather unlikely. To obtain direct evidence for the physiological function of δOAT, we identified and characterised loss-of-function T-DNA insertion mutants. We found that the T-DNA insertion lines SALK_033541 (*oat1*) and SALK_106295 (*oat3*) carry inverted tandem repeats of the T-DNA in the 1^st ^intron and 4^th ^exon of *δOAT*, respectively (Fig. 2A). Segregation analysis confirmed the absence of further T-DNA insertions in *oat1 *and *oat3 *after repeated backcrossing to wildtype Col-0 (data not shown). Plants homozygous for the T-DNA insertions were identified by PCR on genomic DNA (Fig. 2B). In both lines, the T-DNA insertion resulted in the complete loss of transcript accumulation as demonstrated by northern blot analysis (Fig. 2C–D). The probe used covers 351 bp of the conserved domain of pyridoxal-dependent aminotransferases and did not detect any native or truncated transcripts in both lines, thus excluding the translation of any active protein from the *δOAT *gene (Fig. 2A and Additional file 1). In transgenic lines expressing the *δOAT-GFP *fusion construct, the *δOAT *probe detected the native mRNA and a band with higher molecular weight corresponding to the *δOAT-GFP *transcript. Expression of *P5CS1*, the gene responsible for stress-induced Pro biosynthesis, was unchanged in *oat *mutants and *δOAT-GFP *transgenic plants (Fig. 2D). A third line, SALK_010095 (*oat2*), carried the insertion 4 bp upstream of the transcription start site that was determined by [20]. *δOAT *transcripts of the native size were detected in *oat2*, although they were slightly less abundant compared to the wildtype Col-0 (data not shown). Thus the *oat2 *mutant was not included in further studies.

Analysis of genomic sequences revealed no other candidate genes for OATs in Arabidopsis. Still, it was important to analyse OAT activity in the *oat1 *and *oat3 *knockout mutants. In whole plant extracts of 2-week-old wildtype seedlings, a weak but significant OAT activity was detected (Fig. 2F). In *oat1 *and *oat3 *extracts, OAT activity was not significantly increased over control values and accounted for maximally 1/10 of the wildtype activity. Two *δOAT-GFP *expressing lines had 8.5 and 20.4-fold higher OAT activities than the wildtype. Homozygous plants of both *oat1 *and *oat3 *mutant lines showed no obvious phenotypical differences from the wildtype under greenhouse conditions, demonstrating that δOAT-activity is not essential for the normal life cycle of Arabidopsis (data no shown).

**Figure 3:**
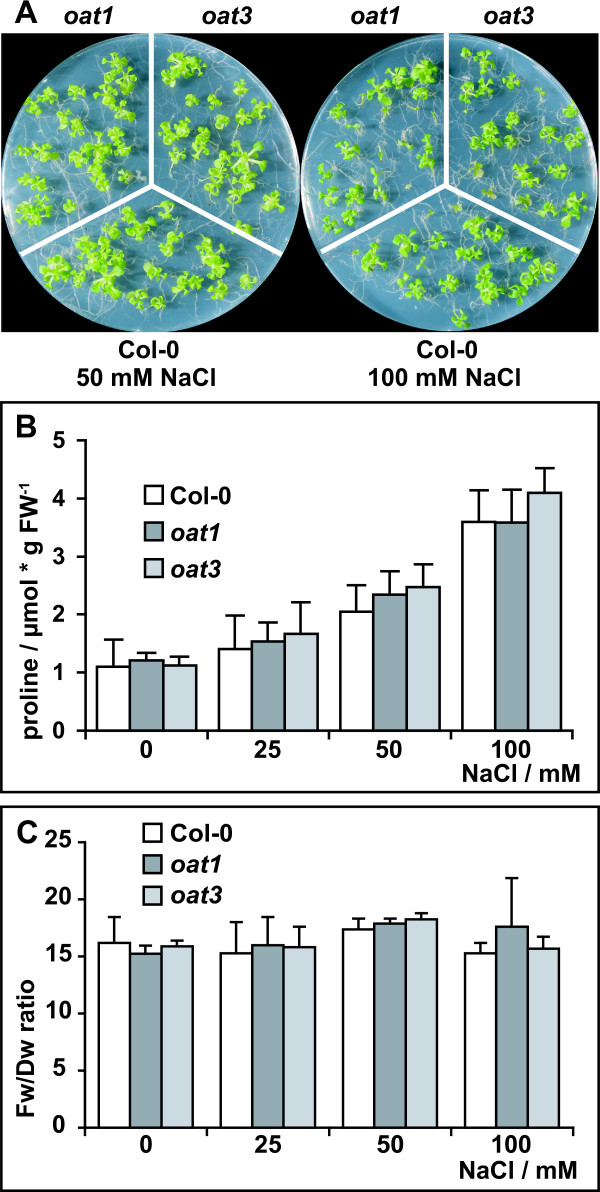
***oat *mutants display the same salt stress responses as wildtype plants**. **A**: Col-0 wildtype, *oat1 *and *oat3 *were grown for three weeks in sterile culture on MS medium supplemented with 60 mM sucrose and increasing amounts of NaCl. **B**: Free Pro levels in 3-week-old plants. **C**: Fw/Dw ratios of plants cultivated under the same conditions. Columns represent the average of 3 (C) or at least 4 (B) independent biological replicates, error bars indicate SD.

To investigate a potential function of δOAT in stress-induced Pro accumulation, we cultivated wildtype, *oat1 *and *oat3 *in sterile culture on media containing increasing amounts of NaCl (Fig. 3A). The mutants displayed similar sensitivity towards NaCl as the wildtype and seedling establishment was almost completely blocked in all three genotypes by the addition of more than 100 mM NaCl. Quantification of free Pro content in 3-week-old plants revealed no significant differences between wildtype and *oat *mutants, neither under control conditions nor after salt stress (Fig. 3B). In all three genotypes the content of free Pro was increased approximately 3-fold by the addition of 100 mM NaCl. Similar Fw/Dw ratios in wildtype and *oat *mutants under all salt concentrations further supported an equal stress tolerance in both genotypes (Fig. 3C). These findings indicate that δOAT does not contribute significantly to stress-induced Pro biosynthesis *in vivo *during salt stress. Additional evidence against a direct entry of Orn-derived P5C into Pro biosynthesis was derived from public microarray-expression data analysed with the BAR e-northern web-tool [28,29]. Over a large set of stress experiments, *δOAT *mRNA levels are in much closer correlation to *P5CDH *mRNA than to *P5CR *mRNA (data not shown).

### Ornithine-δ-aminotransferase is required for utilisation of arginine and ornithine

**Figure 4:**
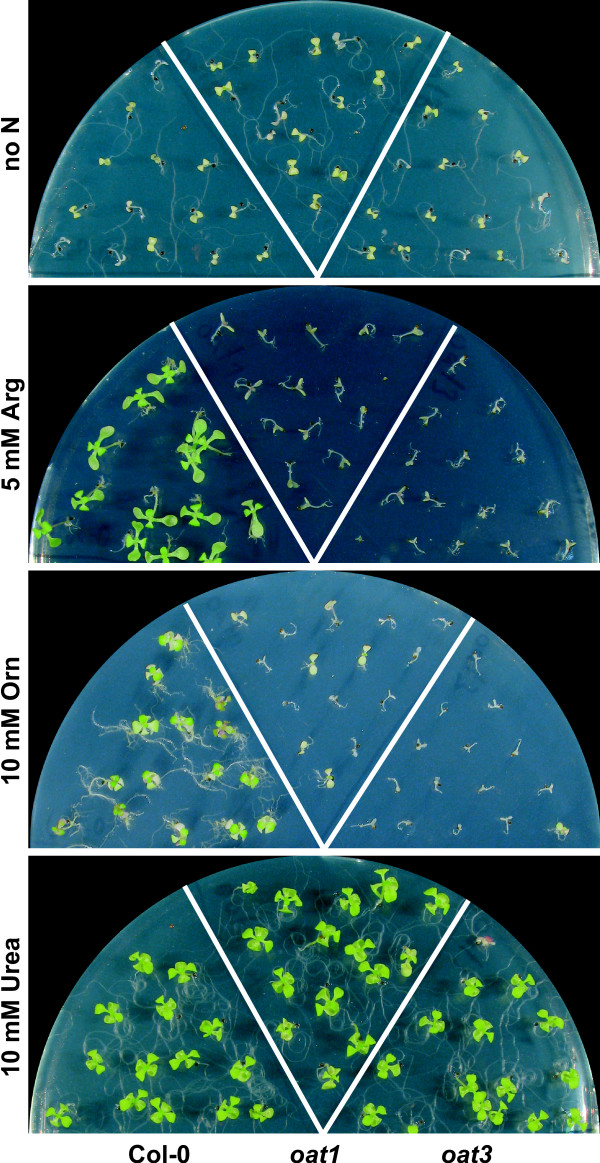
***oat *mutants are unable to use Arg or Orn as nitrogen source**. Col-0 wildtype, *oat1 *and *oat3 *were cultivated on MS medium lacking mineral nitrogen but supplemented with 30 mM sucrose and the indicated concentrations of organic nitrogen sources. Plates without nitrogen, with 5 mM Arg or 10 mM urea were photographed after 4 weeks, the picture of the plate with 10 mM Orn was taken after 6 weeks of growth.

Since the predominant function of δOAT was apparently not in Pro biosynthesis, we considered alternative metabolic functions for this enzyme. Co-localisation with the Arg-breakdown pathway in mitochondria suggested a putative function of δOAT in recycling of nitrogen stored as Arg. To test if δOAT functions in Arg catabolism, we grew wildtype and *oat *mutant seedlings in sterile culture with Arg, Orn or urea as the sole source of nitrogen (Fig. 4). In the absence of any external nitrogen, both wildtype and mutants showed root growth and expanded, de-etiolated cotyledons, but further development was not possible. Arg supported growth of the wildtype, although the plants grew slower when compared to plants grown on normal MS mineral medium. *oat *mutants germinated, but failed to de-etiolate, initiate root growth or develop true leaves on 5 mM Arg as the only nitrogen source. With 10 mM Orn as the only nitrogen source, growth of the wildtype was even more retarded and *oat *mutants were arrested in development at the same stage as on Arg-containing plates. Urea could be used equally well by all three analysed genotypes. These findings demonstrated that *oat *mutants could not use Arg or Orn as nitrogen sources for growth. Comparison with seedlings grown in the absence of nitrogen indicated that supply of Arg or Orn inhibited seedling establishment and use of internal nitrogen reserves in *oat *mutants.

**Figure 5:**
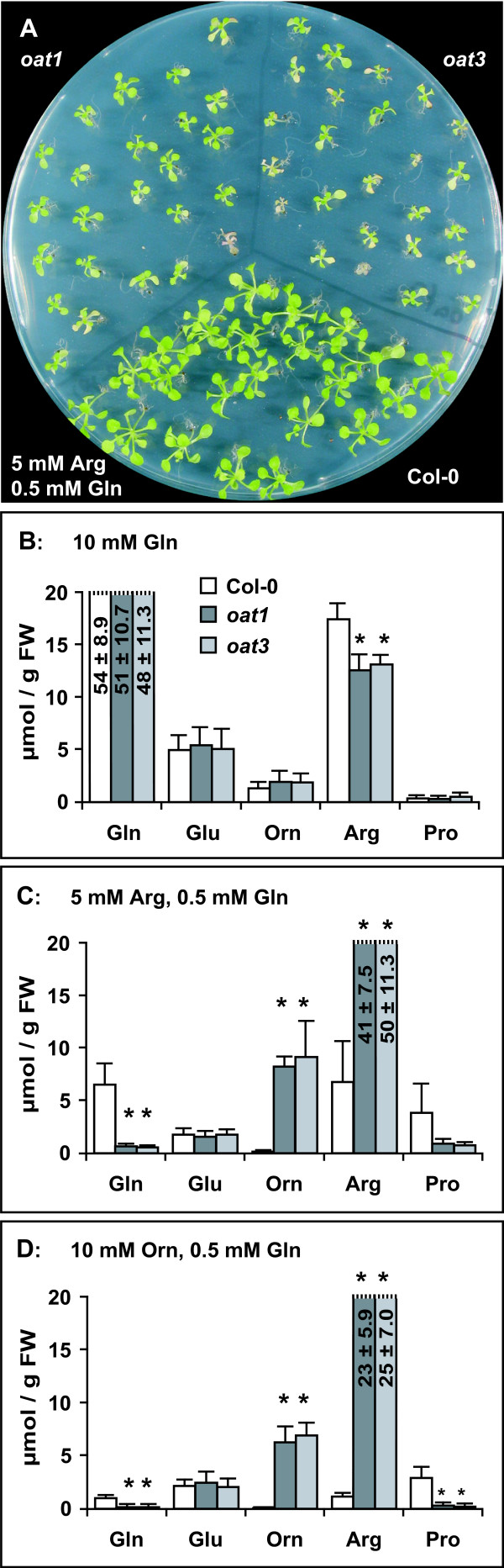
**Metabolism of Arg and Orn is impaired in *oat *mutants**. Col-0 wildtype, *oat1 *and *oat3 *were cultivated for 3 weeks on MS medium lacking mineral nitrogen but supplemented with 30 mM sucrose, 0.5 mM Gln and an additional organic nitrogen source corresponding to 20 mM nitrogen. **A**: Addition of 0.5 mM Gln to 5 mM Arg allowed establishment and limited growth of *oat *mutant seedlings. **B-D**: Profiles of the major free amino acids in excised rosettes of plantlets cultivated on the indicated nitrogen sources. Values are the average of 3 to 4 independent biological replicates, error bars indicate SD. Asterisks indicate significant differences from the wildtype Col-0 (p ≤ 0.05). For the full amino acid profiles see Fig. 6.

A general inhibitory effect of single amino acids to plant cell growth had been observed earlier and could in the case of Arg be abolished by addition of glutamine (Gln) [30]. Indeed, addition of 0.5 mM Gln to 5 mM Arg improved growth and development of both wildtype and *oat *mutants (Fig. 5A). However, *oat *mutants remained chlorotic and grew worse than in the presence of 0.5 mM Gln alone (data not shown). 10 mM Gln as the only nitrogen source enabled much faster growth of Arabidopsis than 5 mM Arg or 10 mM Orn, each supplemented with 0.5 mM Gln. *oat *mutants grew equally well as the wildtype on 10 mM Gln. These findings indicated that inhibitory effects of Orn and Arg were overcome by Gln, but *oat *mutants were not or only poorly able to utilise Arg or Orn as nitrogen sources.

### *oat *mutants accumulate urea cycle intermediates when supplied with arginine

**Figure 6:**
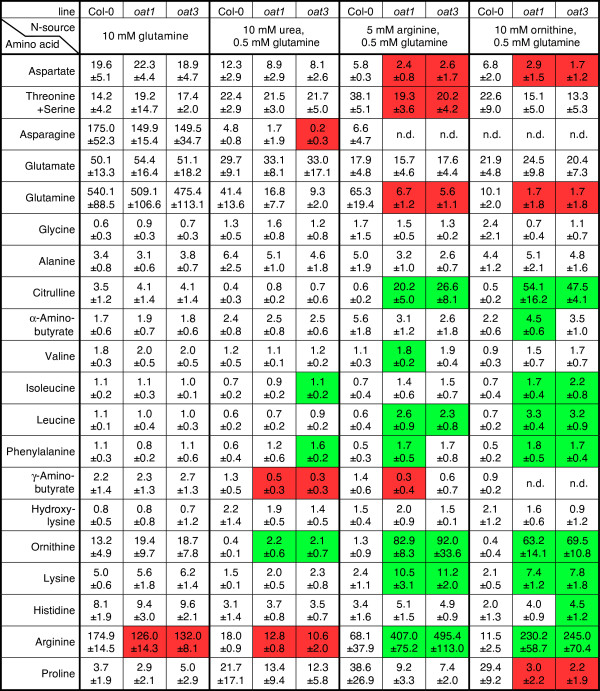
**Amino acids profiles of *oat *mutants grown on different nitrogen sources**. Contents of free amino acids were determined by HPLC. For cultivation conditions see legend to Fig. 5 and the methods section. Amino acid contents are given in μmol/10 mg FW. Values are the mean ± SD of 3 to 4 independent replicates. n.d. = not detected, also not or not consistently detected were cysteine, methionine, tryptophan and tyrosine. Green and red boxes indicate values significantly higher or lower than the wildtype, respectively (p ≤ 0.05 by students t-test).

To determine the fate of externally supplied Arg and Orn in *oat *mutants and wildtype, we determined the pools of free amino acids in seedlings cultivated on Gln, Arg, Orn or urea as nitrogen sources. To support formation of sufficient amounts of biomass in *oat *mutants, 0.5 mM Gln was added to all plates. As expected, free Gln accumulated in plants cultivated on 10 mM Gln, while most other amino acids were present at similar levels as in plants cultivated on 20 mM mineral nitrogen (Fig. 5B, Fig. 6, and data not shown). A slightly reduced Arg content was the only significant difference to the wildtype in *oat *mutants on 10 mM Gln. With urea, Arg or Orn as the main nitrogen source, free Gln levels were progressively lowered and the *oat *mutants always displayed lower Gln content than the wildtype, although differences were only significant on 5 mM Arg (Fig. 5C,D and Fig. 6). With Orn as the main nitrogen source, *oat *mutants were depleted of Gln almost to the detection limit, despite the presence of 0.5 mM Gln in the medium. Interestingly, Glu levels were nearly constant under all conditions analysed and in all genotypes. Levels of asparagine and aspartate basically mirrored the trend of Gln and Glu contents on a lower level. On 10 mM urea as the main nitrogen source, levels of free amino acids were generally low. Significant differences between the wildtype and the *oat *mutants were only observed for γ-aminobutyrate, Arg (both lower in *oat *mutants) and Orn (higher in *oat *mutants). The most striking differences between the wildtype and the *oat *mutants were observed when Arg or Orn were supplied externally. Under these conditions, *oat *mutants accumulated Orn, citrulline (Cit) and Arg. Cit and Orn levels were 34 to 163-fold higher in *oat *mutants than in the wildtype, whereas Arg was increased 6 to 21-fold. Also for leucine, isoleucine, phenylalanine and lysine significant, although smaller, increases were observed. Gln, aspartate and Pro were the only amino acids for which significantly lower levels were observed in *oat *mutants cultivated on Orn or Arg. Based on these amino acid profiles, we conclude that δOAT constitutes a major and possibly the only exit route of nitrogen from Orn or Arg. Accumulation of Cit indicated that Orn and Arg were metabolised after uptake, most likely by enzymes of the urea cycle.

### *oat *mutants are rescued by expression of an δOAT-GFP fusion protein

**Figure 7:**
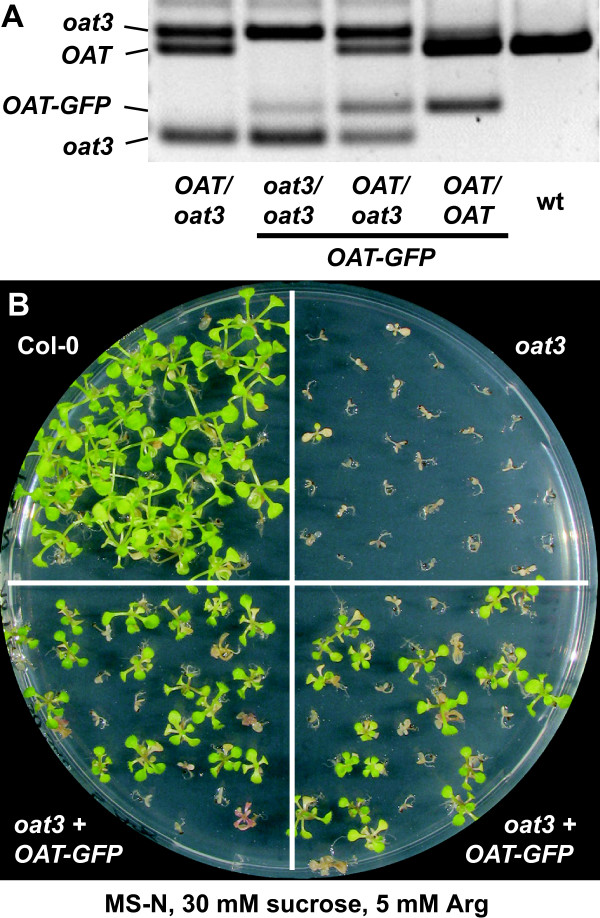
**Complementation of the *oat *mutant phenotype by expression of the Oat-GFP fusion protein**. **A**: Genotyping of the F2-progeny of a cross between *oat3 *and a δ*OAT-GFP *transgenic line. **B**: The capability to utilise Arg as the only nitrogen source is segregating in the progeny of two plants homozygous for the *oat3 *T-DNA insertion but heterozygous for the δ*OAT-GFP *construct.

To demonstrate that the mutant phenotypes of the *oat *knockout mutants are solely based on the lack of δOAT activity, we crossed the *oat3 *mutant with a δOAT-GFP expressing transgenic line with a single T-DNA insertion and clearly visible GFP expression in the T2 and T3 generation. PCR based genotyping of the F2 generation after crossing was used to identify plants homozygous for the *oat3*-T-DNA that additionally carried the *δOAT-GFP *construct (Fig. 7A). Among the progeny of a homozygous *oat3 *plant heterozygous for the δ*OAT-GFP *construct, 39 out of 70 seedlings were scored Arg catabolism positive by expanded, de-etiolated cotyledons and true leaf formation (Fig. 7B). All 39 showed clear GFP expression. Among the 31 Arg sensitive seedlings, 18 did not show any GFP fluorescence, whereas 13 showed expression, mostly with a patchy pattern of GFP-expressing and non-expressing cells. Progeny of a plant homozygous for *oat3 *and the δ*OAT-GFP *construct had even fewer GFP expressing cells and were not able to grow with Arg as the sole nitrogen source, indicating the activation of gene silencing by the combination of the *oat3 *insertion with δ*OAT-GFP *overexpression (data not shown). Rescue of the *oat *mutant phenotype by the GFP fusion protein provided additional evidence that the degradation of Arg for nitrogen recycling requires mitochondrial δOAT activity.

## Discussion

### δOAT is not required for salt-stress induced proline biosynthesis

Like the majority of plants analysed so far, Arabidopsis reacts to high salinity stress by osmotic adjustment accompanied by Pro accumulation. Pro accumulation is the cumulative result of induced biosynthesis, reduced degradation and intercellular re-allocation via specific Pro transport proteins [16,31]. The main source of stress induced Pro biosynthesis is the cytosolic pathway from Glu via GSA/P5C involving the enzymes P5CS and P5CR.

**Figure 8:**
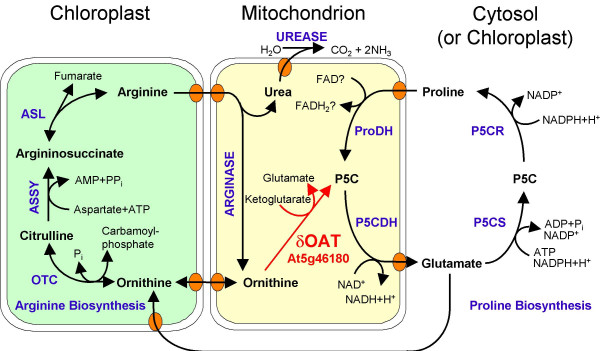
**Compartmentation of Arg and Pro metabolic pathways**. δOAT links the degradation pathways for Arg and Pro, which converge at the level of P5C in mitochondria. Pro biosynthesis occurs in the cytosol or, during stress, in plastids, whereas Arg biosynthesis is constitutively localised in plastids. For details on Arg biosynthesis up to Orn see [3]. ASL: argininosuccinate lyase; ASSY: argininosuccinate synthetase; OTC: ornithine transcarbamylase, P5C: pyrroline-5-carboxylate, P5CDH: P5C dehydrogenase; P5CR: P5C reductase; P5CS: P5C synthetase, ProDH: Pro dehydrogenase.

In bacteria and mammals, transamination of Orn constitutes an alternative route for GSA/P5C and subsequently Pro formation [17]. Recovery of radioactive Pro after feeding of labelled Orn to plants has led to the concept that a similar pathway exists in higher plants [17,25]. However, the exact biochemical pathway and contributing enzymes are subject to controversial debate. While the majority of publications assume that δOAT produces GSA from Orn, which spontaneously forms P5C and is then converted to Pro by P5CR, this hypothesis neglected the localisation of both enzymes to different compartments (Fig. 1 and Fig. 8). In favour of this concept, transgenic plants overexpressing δOAT had higher Pro contents [24,25]. To date, the exact source of Pro accumulating in these δOAT overexpressors has not been determined. We demonstrated here that two T-DNA insertion mutants lacked detectable *δOAT *expression and showed insignificant P5C production from Orn and α-ketoglutarate in whole seedling protein extracts. Both *oat *mutants were not affected in Pro accumulation under stressed or non-stressed conditions.

Additionally, a mitochondrial localisation of δOAT had been predicted before and was confirmed in this study by analysis of plants expressing a δOAT-GFP fusion protein. P5C produced by δOAT inside mitochondria is most probably further converted to Glu by mitochondrial P5CDH. Due to the chemical instability of GSA/P5C, export from mitochondria seems unlikely but can currently not be fully excluded. P5C stimulated O_2 _uptake of isolated intact mitochondria, but very little P5C was produced from Orn or Pro [32]. Orn- or Pro-dependent P5C production and P5C-dependent NAD reduction were measurable only after swelling of mitochondria in low osmolarity buffer, which was attributed to the disruption of ProDH-P5CDH and δOAT-P5CDH enzyme complexes. The impact of swelling on the permeability of the mitochondrial membranes for P5C was not analysed. In δOAT overexpressing plants, non-complexed δOAT could indeed lead to the release of P5C from mitochondria and subsequent conversion to Pro by cytosolic P5CR. Alternatively, the use of the Arabidopsis *δOAT *gene for overexpression in tobacco or rice could have resulted in incomplete import into mitochondria and thus cytosolic δOAT-activity.

Evidence against a role of δOAT in the conversion of Orn to Pro had already come from tracing experiments using differentially labelled ^14^C/^3^H-Orn [23]. Only when the δ-amino group of Orn was labelled with ^3^H, substantial ^3^H activity could be recovered in the Pro fraction. These findings are consistent with the activity of a putative α-aminotransferase that would produce pyrroline-2-carboxylate as an intermediate, or an Orn-cyclodeaminase, which would produce Pro directly. However, long incubation times and possible isotope discrimination effects do not allow excluding the participation of δOAT completely [17]. ^3^H labelled Pro could have also been formed from ^3^H Glu that was formed when δOAT transferred the labelled amino group to α-ketoglutarate. Feeding radioactive Arg or Orn to control or wilted barley leaves indicated that the Orn to Pro conversion was not enhanced by water deficit and that the C-skeleton of Arg contributed maximally 1% of the accumulating Pro [33,34]. These findings are in line with our observation that δOAT deficient mutants retain unchanged levels of salt stress-induced Pro accumulation (Fig. 2). We propose that under normal physiological conditions Orn can be converted to Pro only via Glu, while this conversion is not contributing substantially to stress-induced Pro accumulation. In addition, *oat *mutants provide an excellent tool to investigate if mitochondrial Orn (e.g. from Arg degradation) or externally supplied Orn can be converted to Pro by alternative pathways. Absence of significant amounts of colour development in our OAT assay with *oat *mutant extracts indicated that such alternative pathways are not catalysed by soluble proteins or require different substrates and cofactors. Alternatively, the expression could be too low in young seedlings.

### δOAT constitutes an essential exit route for nitrogen from the urea cycle

Having dismissed the most popular hypothesis for the physiological function of δOAT, we set out to analyse an alternative function in Arg degradation. Arg is effectively taken up from the medium by Arabidopsis roots and distributed to aboveground organs, presumably via transporters of the LHT rather than AAP subfamilies of broad specificity amino acid permeases [35,36]. The first step of Arg breakdown is the cleavage into Orn and urea by arginase, which is localised in mitochondria in plants [9]. Urea can be further degraded by cytosolic urease, and urea supported growth of *oat *mutants and the wildtype equally well (Fig. 4). No information is currently available on the export of urea from mitochondria [10]. However, further catabolism of Orn, the second product of arginase, seems to depend on δOAT activity since neither Arg nor Orn supported growth of *oat *mutants. Instead, intermediates of the urea cycle accumulated to high amounts, indicating that δOAT is required for Arg catabolism and nitrogen recycling (Fig. 5 and Fig. 6). Other metabolites that can be produced from Arg are polyamines, but apparently these are not metabolised further or the capacity of this pathway is too low to supply enough nitrogen to meet the demand of growing *oat *mutant seedlings.

Evaluation of microarray expression data using the BAR eFP-Browser revealed strongest expression of *δOAT *in senescing rosette leaves, floral organs and mature and imbibed seeds [37]. Within the developing embryo, strong *δOAT *expression was detected in cotyledons. These data further support a function of δOAT in storage mobilisation during early seedling development and in nitrogen recovery during senescence.

### Amino acid interconversions and distribution

Orn was less effective than Arg in supporting growth of wildtype Arabidopsis seedlings, which was reflected in generally lower amino acid contents in plants cultured on Orn as the main nitrogen source (Fig. 4 and Fig. 6). This difference to Arg supply could arise from lower uptake rates or impaired inter- or intracellular distribution of Orn. Orn is synthesised in plastids, where it is also further converted to Arg, whereas production of Orn during Arg degradation occurs in mitochondria. Thus high rates of intracellular Orn transport and the occurrence of high Orn concentrations in the cytosol are unlikely to happen under natural conditions. Two members of the mitochondrial carrier protein-family, AtBac1 and AtBac2, were shown to mediate transport of Arg and Orn along with other basic amino acids [11,12]. Both transporters were able to complement a yeast strain deficient in the mitochondrial Orn/Arg transporter Arg11, suggesting mitochondrial localisation also in plants. The high levels of Cit and Arg in *oat *mutants cultivated on Orn suggested import of Orn into plastids (Fig. 5 and Fig. 6). A reversed reaction of arginase is thermodynamically unfavourable and could not be observed with purified enzyme preparations even in the presence of both Orn and urea in high concentrations [38]. Orn to Cit conversion was previously observed in purified mitochondria and could constitute an alternative pathway to direct Orn import into plastids [39]. High levels of Cit after Arg feeding of *oat *mutants indicate that Orn originating from Arg breakdown is terminally converted to Cit inside mitochondria or is transferred from mitochondria to plastids. Substantial production of Cit by reversion of the argininosuccinate synthetase reaction from the Arg biosynthesis pathway is unlikely due to the low pyrophosphate levels in plastids [40,41]. Synthesis of Arg from Orn requires two atoms of nitrogen per molecule of Arg, thus requiring net N-input in case of Orn feeding. This is consistent with the extreme Gln depletion of *oat *mutants fed with Orn (Fig. 5 and Fig. 6).

Irrespective of the nitrogen source provided, *oat *mutants had an increased content in Orn, indicating that catabolism of Arg is constitutively operative in wildtype plants. Surprising were the decreased levels of Arg in *oat *mutants grown on Gln or urea (Fig. 5 and Fig. 6). Arg biosynthesis is subject to feedback inhibition by the end product at the level of N-acetyl glutamate kinase, which catalyses the key regulatory step of Arg biosynthesis [3]. Arg mediated inhibition of N-acetyl glutamate kinase can be alleviated by the plastidic PII protein, but the precise role of this interaction in regulating Arg biosynthesis is yet unknown [42]. The block in mitochondrial Arg catabolism in *oat *mutants potentially leads to an altered C/N ratio in plastids or a localised increase in Arg concentrations, which in turn could reduce the total rate of Arg biosynthesis. Recently a genetically encoded nanosensor for Arg was developed, that can be used to report cytosolic, mitochondrial or plastidic Arg levels in wildtype and *oat *mutants under various nutrition regimes [43].

Also the significant increase in the contents of leucine, isoleucine, phenylalanine and lysine in Orn-fed, and partially also in Arg-fed, *oat *mutants indicated disturbances of amino acid metabolism. All increased amino acids have high C/N ratios, consistent with a deficiency of *oat *mutants to mobilise nitrogen from Orn and Arg.

The complete degradation of Arg via arginase, δOAT and P5CDH yields two molecules of Glu per molecule of Arg. Despite the consequential massive differences in mitochondrial Glu production between wildtype and *oat *mutants grown on Arg or Orn, Glu levels were the same in both genotypes (Fig. 5 and Fig. 6). Similar Glu homeostasis was observed in many studies on nitrogen nutrition, environmental stress or mutant analyses and was proposed to indicate a special regulatory function of Glu levels [44]. The insensitivity of Glu levels to the deletion of δOAT is presumably the prerequisite for the unchanged capacity of *oat *mutants to accumulate Pro under stress conditions.

## Conclusion

Decades of biochemical analyses have produced the basis for our understanding of plant primary metabolism and are now complemented by genomic, proteomic and metabolomic approaches. Still, the compartmentation of metabolic processes to specific organelles or protein supercomplexes is far from being fully uncovered. Determination of the exact role of a specific enzyme in the metabolic and regulatory networks of plant cells still requires careful and thorough gene for gene analysis. We show here that Arg and Pro catabolism are co-localised in mitochondria and converge in the formation of GSA/P5C, which is further metabolised to Glu by P5CDH. The detection of unchanged Pro levels in Arabidopsis *oat *mutants provides strong evidence against a shortcut from Arg catabolism to Pro synthesis that bypasses Glu and cytosolic P5CS activity. It remains to be investigated if other plant species with more than one *P5CDH *or *OAT *gene have differently localised isoforms and thus other metabolic possibilities.

## Methods

### Plant material and growth conditions

Arabidopsis (*Arabidopsis thaliana *(L.) Heynh. ecotype *Col-0*) and T-DNA insertion lines SALK_033541 (*oat1*), SALK_010095 (*oat2*) and SALK_106295 (*oat3*) were obtained from the NASC. Presence of the T-DNA and allelic status was verified by PCR and sequencing of the T-DNA flanking sequences. Gene specific primers were: Oat-f: 5'agtcttggattaacttaggagag, Oat-r: 5'gtcccatatagttgagccattc for *oat1 *and *oat2*; Oat-f2: 5'gctttcatggacgtacattag, Oat-r2: 5'caagtatcaccatgtcaggac for *oat3*; the T-DNA left border specific primer was 5'ttcggaaccaccatcaaacag. None of the three mutant lines expressed clear kanamycin resistance. All physiological experiments were performed with homozygous progeny of plants backcrossed three times to *Col-0*. Plants were cultivated axenically in 9 cm Petri dishes on commercial MS medium (Duchefa, Netherlands) or self-made MS medium, in which KNO_3 _and NH_4_NO_3 _were replaced by 20 mM KCl [45]. Media were supplemented with sucrose and nitrogen containing compounds as indicated for each experiment and solidified with 8 g/l purified agar (BD biosciences, San Jose, CA, USA). Seeds were surface sterilised by sequential treatment with 70% (V/V) EtOH and 1% (W/V) NaOCl/0.01% (V/V) Triton-X-100 and vernalised for 24 h at 4°C in 0.1% (W/V) agarose. Plants were cultivated in an air-conditioned room with short day (9 h) light period and a light intensity of 110 μmol photons*s^-1^*m^-2 ^from mixed fluorescence tubes (Osram, Germany) at a constant temperature of 22°C. For the OAT activity assay, plants were cultivated under constant agitation and with 24 h low light in liquid MS medium supplemented with 60 mM sucrose. For seed production, plants were kept in a greenhouse with a light period of at least 16 h. *Nicotiana benthamiana *Domin plants were cultivated on commercial gardening soil in the greenhouse under long day conditions.

### δOAT-GFP construct and imaging

The open reading frame of δ*OAT *was amplified by PCR from EST clone H4E5 (GenBank W43737; ABRC, Ohio) with the primers 5'ctggatccgactctaatggcagccaccac and 5'ctggatccgcatagaggtttcttccac. The resulting PCR product was cloned via the introduced BamHI sites into the vector pEZT-NL (Dave Erhardt, [46]). *Agrobacterium tumefaciens *strain LBA4404 was used for transient transformation of *N. benthamiana *leaves and floral dip transformation of Arabidopsis [47,48]. Protoplasts from transformed leaves were obtained by overnight incubation with cellulase and macerase (Serva, Heidelberg, Germany) and viewed under an Axiovert 200 M epifluorescence microscope (Carl Zeiss, Oberkochen, Germany). Filter sets used for GFP, MitoTracker orange and chlorophyll were 38HE (excitation 470 ± 20 nm, emission 525 ± 25 nm), 43HE (550 ± 12.5/605 ± 35) and 45 (560 ± 20/630 ± 32.5), respectively. Cross-detection of GFP and MitoTracker was negligible. Images were captured with an AxioCam MRm monochrome digital camera. False colouring and overlay of images was performed using AxioVison software.

### RNA isolation and detection

Total RNA was extracted from two-week-old axenically cultured seedlings with Trizol reagent (Invitrogen, USA). RNA was separated by denaturing agarose gel electrophoresis and transferred to a positively charged nylon membrane by capillary transfer. *δOAT *transcripts were detected by hybridization with DIG-labelled PCR products obtained with primers Oat-f2 and Oat-r2 and the cloned cDNA as template, followed by detection with alkaline phosphatase coupled anti-DIG antibodies and the chemiluminescent substrate CDP-star (Roche, Switzerland). *P5CS1 *transcripts were detected with a 185 bp fragment of the 5'UTR amplified and subcloned from genomic DNA.

### OAT activity assay

The assay procedure was a combination of methods described by [49] and [50]. Fresh seedlings from liquid culture were rinsed briefly with distilled water, blotted dry and ground in a mortar in 5 μl/mg Fw ice-cold extraction buffer (100 mM KHPO_4_, 10 mM β-MSH, 1 mM EDTA, 0.2 mM pyridoxal 5'phosphate, pH 7.9). The extract was centrifuged for 15 min at 16400 rpm at 4°C in a tabletop centrifuge and desalted over a 5 ml HiTrap column equilibrated with extraction buffer (GE healthcare, UK). The assay mixture, consisting of 25 mM Orn, 25 mM α-ketoglutarate and 100 μl plant extract in a total volume of 500 μl extraction buffer, was incubated at 37°C for 20 min. The reaction was terminated by the addition of 150 μl 3 M HClO_4_. P5C was detected with 100 μl of 2% (W/V) ninhydrin in water and heating to 96°C for 6 min. The water-insoluble reaction product was extracted with 1 ml toluene and quantified by measuring the absorbance at the maximum of 520 nm. For blanks, HClO_4 _was added before the extracts and processed identically. No P5C was detected when either Orn or extract were omitted. Proline produced a product with an absorbance maximum at 540 nm, which was not observed in the assay. The protein concentration of the extract was determined by a Bradford assay and used to normalise the amount of P5C, which was further converted to arbitrary activity units in which the wildtype activity was set to 1.

### Proline and amino acid determination

Free Pro was quantified by a method modified from [51]: Leaf material was ground in liquid N_2 _with a mortar and pestle and allowed to thaw in 3 μl/mg Fw of 10% (W/V) sulfosalicylic acid. After extraction for at least 30 min on ice, the samples were centrifuged and 250 μl of the supernatant were mixed with 150 μl of HAc and 150 μl of acidic ninhydrin reagent (125 mg ninhydrin in 2 ml 6 M ortho-phosphoric acid and 3 ml HAc) and reacted for 20 min at 96°C. The mixture was cooled on ice and the red reaction product was extracted with 1 ml toluene. Absorbance of the toluene supernatant was read at 520 nm and Pro concentrations were calculated using standard curves from 0 to 10 mM Pro treated in the same way as the samples. Orn is known to give equal absorption values as Pro in this assay, but Orn levels were less than 2% of Pro levels in NaCl stressed or non-stressed wildtype or *oat *mutants cultivated on normal mineral nitrogen sources (data not shown).

Methanol/water extracted amino acids were quantified by HPLC with post-column ninhydrin derivatisation as described in [52].

### Database mining

Data on subcellular localisation prediction were taken from the ARAMEMNON database [22,53]. The transit peptide cleavage site was predicted with TargetP [21,54]. Microarray expression data were analysed with the webtools offered by BAR (The Bio-Array Resource for Arabidopsis Functional Genomics [29])

## Abbreviations

Arg: arginine; ASL: argininosuccinate lyase; ASSY: argininosuccinate synthetase; Cit: citrulline; Dw: dry weight; Fw: fresh weight; Gln: glutamine; Glu: glutamate; GSA: glutamate-5-semialdehyde; δOAT: Ornithine-δ-aminotransferase; Orn: ornithine; OTC: ornithine transcarbamylase; P5C: pyrroline-5-carboxylate; P5CDH: P5C dehydrogenase; P5CR: P5C reductase; P5CS: P5C synthetase; Pro: proline; ProDH: Pro dehydrogenase.

## Authors' contributions

DF designed the study and performed most of the experiments; BS and WK performed amino acid analyses and helped in compiling and interpreting the data. All authors have read and approved the final manuscript

## Supplementary Material

Additional file 1 provides two supplementary figures. Supplementary figure 1 illustrates the localisation of the δOAT-GFP fusion protein in intact Arabidopsis cells. Supplementary figure 2 shows the full picture of the northern blot also shown in Fig. 2C, to demonstrate the absence of truncated δ*OAT*-specific transcripts. Click here for file
