# Supplementary material for: Ornithine-δ-aminotransferase is essential for Arginine Catabolism but not for Proline Biosynthesis
Source: BMC Plant Biol. 2008 Apr 17;8:40. doi: 10.1186/1471-2229-8-40 (PMC2377265; doi:10.1186/1471-2229-8-40)
Supplement: Additional file 1 — provides two supplementary figures. Supplementary figure 1 illustrates the localisation of the δOAT-GFP fusion protein in intact Arabidopsis cells. Supplementary figure 2 shows the full picture of the northern blot also shown in Fig. 2C, to demonstrate the absence of truncated δOAT-specific transcripts. [file 1471-2229-8-40-S1.pdf]

Supplementary figures 1 & 2, Funck et al. 2008

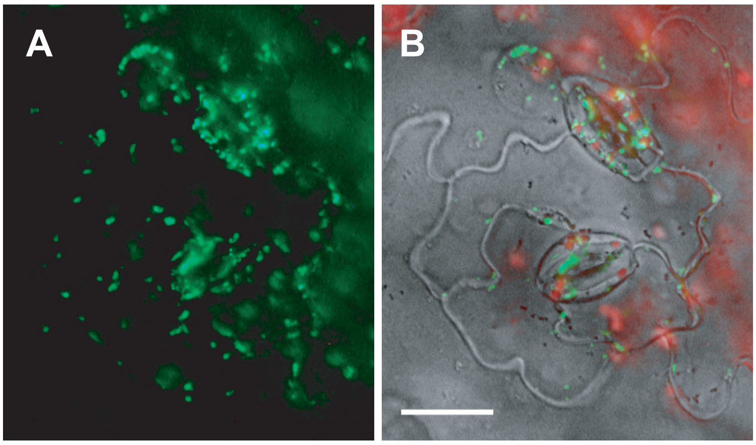

Suppl. Fig. 1: δOAT localisation in epidermis and guard cells
A hand section of the lower epidermis of a mature leaf stably expressing a δOAT-GFP fusion construct under control of the CaMV 35S promoter. **A**: GFP fluorescence; **B**: Overlay of a brightfield image of the same area as A with GFP and chlorophyll fluorescence; scale bar = 20 μm

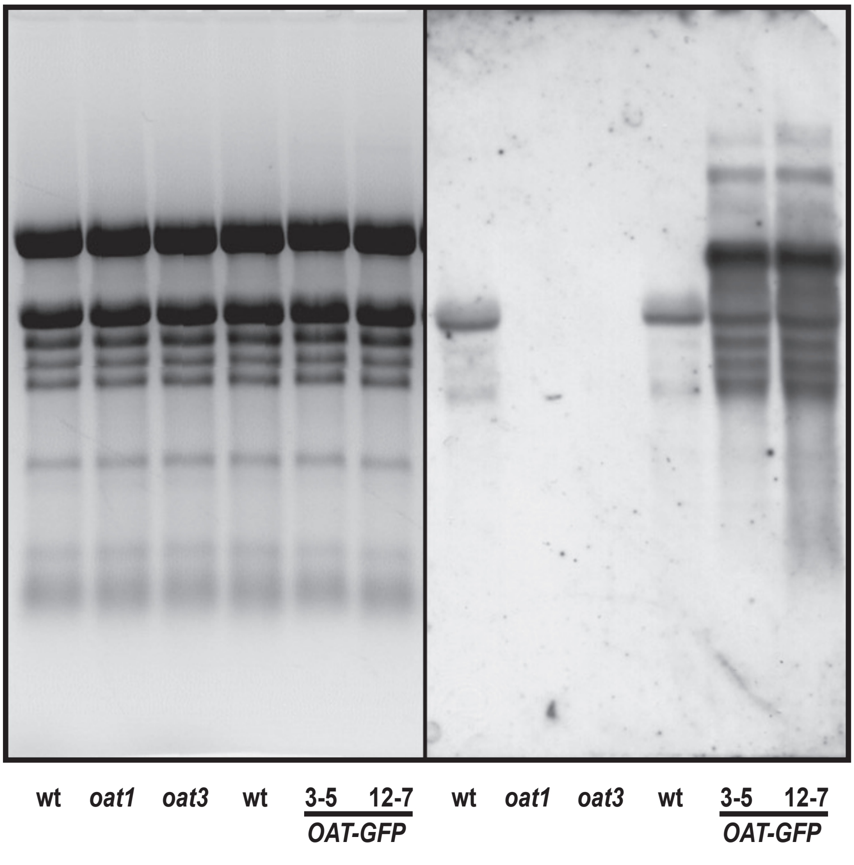

Suppl. Fig. 2: δ*OAT*-transcripts are not detectable in *oat1* and *oat3*
Ethidium bromide stain of total RNA (left) and detection of δ*OAT*-specific transcripts (right).
